# Sensitivity of anti-SARS-CoV-2 serological assays in a high-prevalence setting

**DOI:** 10.1007/s10096-021-04169-7

**Published:** 2021-02-03

**Authors:** Lisa Müller, Philipp Niklas Ostermann, Andreas Walker, Tobias Wienemann, Alexander Mertens, Ortwin Adams, Marcel Andree, Sandra Hauka, Nadine Lübke, Verena Keitel, Ingo Drexler, Veronica Di Cristanziano, Derik Franz Hermsen, Rolf Kaiser, Friedrich Boege, Florian Klein, Heiner Schaal, Jörg Timm, Tina Senff

**Affiliations:** 1 Institute of Virology, University Hospital Düsseldorf, Heinrich Heine University Düsseldorf, 40225 Düsseldorf, Germany; 2 Institute of Medical Microbiology and Hospital Hygiene, Heinrich Heine University Düsseldorf, 40255 Düsseldorf, Germany; 3 Department of Gastroenterology, Hepatology and Infectious Diseases, University Hospital Düsseldorf, Heinrich Heine University Düsseldorf, 40255 Düsseldorf, Germany; 4 Institute of Virology, Faculty of Medicine and University Hospital of Cologne, University of Cologne, 50935 Cologne, Germany; 5 Institute of Clinical Chemistry and Laboratory Diagnostics, Medical Faculty, University Düsseldorf, 40255 Düsseldorf, Germany

**Keywords:** SARS-CoV-2, COVID-19, Serology, Neutralizing antibodies, Immunofluorescence test, Seroprevalence

## Abstract

Evaluation and power of seroprevalence studies depend on the performed serological assays. The aim of this study was to assess four commercial serological tests from EUROIMMUN, DiaSorin, Abbott, and Roche as well as an in-house immunofluorescence and neutralization test for their capability to identify SARS-CoV-2 seropositive individuals in a high-prevalence setting. Therefore, 42 social and working contacts of a German super-spreader were tested. Consistent with a high-prevalence setting, 26 of 42 were SARS-CoV-2 seropositive by neutralization test (NT), and immunofluorescence test (IFT) confirmed 23 of these 26 positive test results (NT 61.9% and IFT 54.8% seroprevalence). Four commercial assays detected anti-SARS-CoV-2 antibodies in 33.3-40.5% individuals. Besides an overall discrepancy between the NT and the commercial assays regarding their sensitivity, this study revealed that commercial SARS-CoV-2 spike-based assays are better to predict the neutralization titer than nucleoprotein-based assays are.

**Supplementary Information:**

The online version contains supplementary material available at 10.1007/s10096-021-04169-7.

## Introduction

In December 2019, a new coronavirus, severe acute respiratory syndrome coronavirus 2 (SARS-CoV-2), emerged in China and its pandemic spread resulted in more than 30 million infected people according to the World Health Organization [1–3]. Sensitive serological SARS-CoV-2 assays are of great importance for seroprevalence studies and retrospective diagnosis of SARS-CoV-2 infections and aide in estimating prevalence and incidence [4]. Additionally, these assays are necessary to identify donors for convalescent plasma therapy and to determine antibody titers to assess induced immunity after vaccination [5]. Here, we assess and compare different commercial serological tests as well as an in-house neutralization and immunofluorescence test (IFT) in the context of a SARS-CoV-2 high-prevalence setting.

## Material and methods

### Patients

Serum samples from 42 non-randomized volunteers from the same local area were collected on April 9, 2020. Individuals had direct or indirect contact to a German index patient with a PCR-confirmed SARS-CoV-2 infection and hospitalization on April 24. Previous SARS-CoV-2 PCR testing and symptoms and their onset were queried. Due to the small cohort size, a classification of the severity of symptoms in PCR-confirmed cases was not performed. Health authorities tested 26 of 42 by PCR before sample collection on April 9 with 8 of 26 being SARS-CoV-2 PCR positive.

### Ethical statement

The study was approved by the local ethics committee (study number: 5350). Written informed consent was given from each included individual.

### Commercially available anti-SARS-CoV-2 test systems

Samples were tested for anti-SARS-CoV-2 antibodies with four commercially available test systems: EUROIMMUN (EI), Roche, Abbott, and DiaSorin, recognizing either SARS-CoV-2 virus spike (S) protein or nucleocapsid (N) antibodies. Euroimmun ELISA directed against the S1 domain of the spike protein detecting IgA and IgG was performed on the Euroimmune Analyzer I-2P according to manufacturer’s instructions. OD ratio ≥1.1 for IgA and IgG was considered positive, ≤0.8 as negative, and ≥ 0.8 ≤ 1.1 as borderline. Upper detection limits were OD ratio = 7 for IgA and OD ratio = 10 for IgG. IgG antibodies against S1/S2 domains of SARS-CoV-2 spike were detected through chemiluminescent immunoassay (CLIA) from DiaSorin on a LIAISONX. SARS-CoV-2 S1/S2 IgG antibody concentrations are given as arbitrary units (AU/ml). Samples <12 AU/ml were interpreted as negative, 12-15 AU/ml as borderline, and ≥ 15 AU/ml as positive. The Elecsys® anti-SARS-CoV-2 electrochemiluminescence immunoassay (ECLIA) from Roche was performed on a cobas e801 immunoassay analyzer for the detection of antibodies (including IgG) against SARS-CoV-2 N antigen. Cut-off was based on the measurement of two calculators, and the result was given as signal sample to cutoff (COI). COI <1.0 is negative for anti-SARS-CoV-2 antibodies and COI ≥1.0 is considered positive. The SARS-CoV-2 IgG chemiluminescent microparticle immunoassay (CMIA) from Abbott performed on an ARCHITECT i2000 SR detects IgG antibodies to N. The relation of chemiluminescent RLU and the calibrator is given as the calculated index (S/C). An index (S/C) ≥1.4 is considered positive and <1.4 as negative.

### Cell culture

Vero cells (ATCC-CCL-81 obtained from LGC Standards) were grown in Dulbecco’s modified essential medium (DMEM) with 1% penicillin and streptomycin (Gibco, 100 U/ml penicillin and 100 μg/ml streptomycin) and 2% fetal calf serum (FCS, PAN Biotech) and were cultured in a 5% CO_2_ humified atmosphere at 37 °C.

### SARS-CoV-2 virus isolate

For the neutralization test, SARS-CoV-2 isolate NRW-42 was used [6]. The complete sequence of this virus isolate is online (EPI_ISL_425126). There is a single-nucleotide exchange in the spike ORF between the Wuhan-Hu1 reference sequence and the NRW-42 sequence used for NT and IFT. The isolate carries a A>G mutation within the Spike gene at nucleotide position 23,403 which is located within the S1 domain, but outside of the RBD or RBM motif respectively. There is no nucleotide exchange in the nucleocapsid ORF. Unfortunately, antigenic identity of sequences used in the commercial tests is unavailable.

### Neutralization test

To detect SARS-CoV-2-neutralizing antibodies, a modified neutralization test was performed [7]. Sera were heat inactivated for 30 min at 56 °C and briefly centrifuged. Initial 1:5 dilutions were prepared in duplicate per patient followed by twofold serial dilutions performed in 50-μl volume with DMEM (1% penicillin and streptomycin, 2% FCS). A total of 50 μl of SARS-CoV-2 stock dilution (final conc. TCID_50_ of 50) was added to the sera dilutions, control sera, and virus only controls (no serum added). Cell-free plates were pre-incubated at 37 °C for 1 h. Afterwards, 100 μl of cell suspension containing 7 × 10^4^/ml Vero cells was added to samples and cell growth controls. Plates were incubated for 4 days. By microscopic inspection, the titer of neutralizing antibodies was determined as the highest serum dilution without a cytopathic effect (CPE). The reciprocal of the serum dilution is given as the NT titer. A neutralization titer of ≥20 was considered positive. Samples from three individuals with documented coronavirus HCoV-229E, HCoV-OC43, and HCoV-NL63 infections served as controls for cross reactivity (NT titer = 0).

### Immunofluorescence test

Vero cells were seeded at a density of 10^4^ cells per well into a 48-well plate. After 24 h, cells were infected with SARS-CoV-2 NRW-42 isolate (TCID_50_ of 50) except for controls. At 2 dpi, fixation was performed with ice-cold methanol for 20 min at −20 °C. Subsequently, cells were washed three times with PBS, permeabilized with 0.5% Triton X-100 in PBS for 20 min, and washed again three times. Sera were diluted 1:40 in PBS containing 5% FCS. Cells were incubated in 200-μl patient serum, for 2 h at room temperature. Two SARS-CoV-2-infected wells and one well with uninfected cells were used per patient. Positive control serum was obtained from a patient with high levels of anti-SARS-CoV-2 IgG. After washing, cells were incubated for 1 h at room temperature with anti-human IgG FITC conjugate (Life Technologies, USA) diluted 1:40 in PBS containing 0.1% Evans blue and 5% FCS. Cells were washed three times with PBS and analyzed by microscopy. IFT results were independently evaluated by two staff members. Positive results indicated IFT titer ≥ 40.

### Statistical analysis

GraphPad Prism version 8.0.2 was used for statistical analysis. Pearson correlation analysis was used to assess correlation between serological assays. Good correlation was assumed if *r* ≥ 0.5 and moderate if *r* ≥ 0.3 in combination with *p* ≤ 0.05. One-way analysis of variance (ANOVA) was performed for comparison between groups after checking for normal distribution. The respective *p* values are given as **p ≤ 0.01 and ***p ≤ 0.001.

## Results

### SARS-CoV-2 high-prevalence setting—social and working contacts of a German index patient

On 24 February 2020, a patient from the Heinsberg District, Germany was diagnosed SARS-CoV-2 positive by RT-PCR. By February 28_,_ contact tracing from health authorities identified 37 secondary cases. In addition, this index patient was associated with a super spreading event held on 15 February 2020, and >1000 SARS-CoV-2 cases were linked to this event [8].

To assess different serological tests for detection of anti-SARS-CoV-2 antibodies in the context of a high-prevalence setting, blood samples of 42 social and working contacts of this index patient were collected on April 9, 2020 and subsequently analyzed. Importantly, since the index patient was hospitalized on February 24, contact to this patient must have occurred at least 6 weeks before sample collection. Despite reported symptom onset was around 10 days prior to admission to hospital, the patient continued to actively participate in social and business life.

The study population contained slightly more females than males (26/16 61.9%, 38.1%) and individuals were aged between 18 and 70 years (median 44). Although only eight of the 42 individuals were previously tested positive for SARS-CoV-2-RNA by RT-PCR, 26 described symptoms including fever (38.5%), cough (65.4%), fatigue (50%), shortness of breath, or difficulty of breathing (30.8%) while 16 reported no symptoms (Table S1).

### Determining SARS-CoV-2 seroprevalence by an in-house SARS-CoV-2 immunofluorescence and neutralization test

First, an in-house neutralization test was performed to identify SARS-CoV-2 seropositive individuals in the described study population. The neutralization test, including the cut-off NT titer of ≥20, was previously validated with 30 SARS-CoV-2 negative sera (NT titer <10; PCR negative or sampled before December 2019) and 25 positive sera from RT-PCR-positive individuals (NT titer 20 to 10,240) and resulting NT titers reflected the current literature [9, 10].

**Fig. 1:**
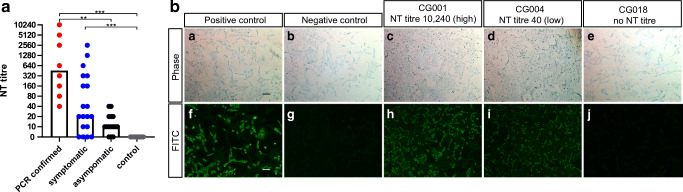
SARS-CoV-2-neutralizing antibodies stratified according to status of study participants and exemplary immunofluorescence test results, Heinsberg District, Germany, April 2020 (*n* = 42). FITC: fluorescein isothiocyanate; NT: neutralization test. **a** Neutralization test results of 42 individuals grouped by their status in PCR confirmed (red), symptomatic (blue), and asymptomatic (black) and 11 control sera from healthy individuals sampled before December 2019. The reciprocal of the NT titer is depicted, and bars represent the respective median. The cut-off was defined as ≥20. One-way ANOVA was used to compare groups (**p ≤ 0.01 and ***p ≤ 0.001). **b** Exemplary anti-SARS-CoV-2 IgG immunofluorescence test results of 3 out of 42 tested individuals. Phase contrast (a−e) and FITC fluorescence detected at 488 nm (f−j). Serum of a severe hospitalized COVID-19 case served as a positive control (a+f) and (b+g) depict the result of a negative control serum. IFT results from a patient with a high NT titer (c+h; NT titer 10,240), a low NT titer (d+i NT titer 40), and no neutralization potential (e+j). Scale bar is 100 μm

Neutralizing antibodies (NT titers ≥20) were detected in 26 of the 42 serum samples (61.9%). Besides the RT-PCR-confirmed SARS-CoV-2 cases (*n* = 8), 13 out of the 19 symptomatic (68.4%) and 5 of the 15 asymptomatic (33.3%) individuals had neutralizing antibodies (Table S2). Neutralizing antibody levels in asymptomatic individuals were significantly lower compared to PCR-confirmed cases (*p* ≤ 0.01, Fig. 1).

To support the NT-based finding of a high SARS-CoV-2 seroprevalence in our study population, an in-house immunofluorescence test (IFT) detecting anti-SARS-CoV-2 IgG was performed. Of the 26 sera positive in the neutralization test, 23 were also positive in the IFT (sensitivity 88.5%, 95% CI [0.710-0.960]). Additionally, negative IFT results were associated with low (≤40) NT titers. This overall supports the finding of a high seroprevalence in our study population as determined by NT.

### Sensitivity of commercial high-throughput SARS-CoV-2 antibody assays

Based on in-house NT results, supported by IFT, anti-SARS-CoV-2 antibodies were found in 26 of the 42 sera. Since both methods are time-consuming and labor-intensive, suitability of antibody testing was analyzed with four different commercially available automated serological test systems targeting either the nucleocapsid protein (N) or the spike protein (S) of SARS-CoV-2 (Table 1, Figure S1/S2).

**Table 1:** Patient characteristics and serological test results of all anti-SARS-CoV-2 assays performed in this study, Heinsberg District, Germany, April 2020 (*n* = 42)

Sample ID	Age in years	Gender	PCR	Status	Reciprocal NT titer	IgG IFT	EUROIMMUN		DiaSorin	Roche	Abbott
							ELISA IgA (OD ratio)*	ELISA IgG (OD ratio)*	CLIA IgG (AU/ml)*	ECLIA (COI)**	CMIA IgG (index S/C)**
CG001	50	m	pos	sym	10,240	++	>7	8,68	80.7	18.8	4.99
CG005	46	f	pos	sym	5120	+	4.36	>10	372	85.1	8.31
CG007	59	m	pos	sym	2560	++	>7	9,55	128	57.1	9.20
CG012	50	f	pos	as	40	+	0.24	0,62	12.2	0.1	0.06
CG015	54	m	pos	sym	320	+	2.11	0,56	16.2	29.2	4.34
CG031	29	m	pos	sym	80	+	3.41	4,84	68.4	55.7	4.72
CG042	24	f	pos	sym	640	+	4.55	6,63	82.8	23.4	3.75
CG043	43	m	pos	sym	160	+	0.4	2,15	63.4	68.1	6.76
CG002	45	f	neg	sym	10	−	0.51	0,16	4.84	<0.1	0.02
CG003	43	m	neg	sym	20	+	0.58	0,39	6.57	4.1	1.44
CG004	43	f	neg	sym	40	+	0.43	0,9	12.6	1	3.69
CG006	55	f	neg	sym	10	−	0.3	0,21	<3.8	<0.1	0.02
CG009	25	m	neg	sym	0	−	0.29	0,22	14.4	<0.1	0.03
CG011	18	f	n/a	sym	0	−	0.33	0,32	10.6	<0.1	0.04
CG013	43	f	n/a	sym	160	+	0.15	0,18	13.3	<0.1	0.02
CG014	55	f	neg	sym	2560	++	1.49	8,66	105	66.9	8.07
CG016	55	f	neg	sym	1280	+	1.19	1,81	30.7	96.1	9.44
CG017	59	m	neg	sym	160	+	0.54	0,97	10.2	22.2	4.60
CG020	38	f	neg	sym	320	+	1.11	7,84	123	75.9	9.03
CG021	39	m	n/a	sym	20	−	0.74	0,24	18.8	<0.1	0.02
CG022	41	m	neg	sym	640	+	0.68	4,47	60.6	91	8.74
CG026	22	f	neg	sym	0	−	0.2	0,29	16.5	<0.1	0.01
CG028	22	f	n/a	sym	0	−	0.18	0,27	11.2	<0.1	0.01
CG032	27	f	n/a	sym	40	+	1.57	3,97	79.5	10.3	2.17
CG033	29	f	n/a	sym	20	+	0.12	0,21	11.2	<0.1	0.12
CG040	23	f	n/a	sym	320	+	1.32	3,26	32.4	12	3.29
CG044	37	f	neg	sym	20	+	1.41	1,04	18	1.5	1.17
CG008	23	f	neg	as	10	−	0.3	0,21	8.15	<0.1	0.06
CG010	30	f	neg	as	10	−	0.38	0,18	6.5	0.1	0.02
CG018	46	m	n/a	as	0	−	0.27	0,15	13.5	<0.1	0.19
CG019	50	f	n/a	as	10	−	0.3	0,31	<3.8	<0.1	0.02
CG023	49	m	n/a	as	10	−	0.45	0,28	11.6	<0.1	0.01
CG024	46	f	n/a	as	10	−	0.19	0,17	<3.8	<0.1	0.03
CG025	70	f	neg	as	40	−	0.19	0,18	10.8	<0.1	0.02
CG027	47	f	neg	as	20	+	0.43	0,15	10.9	<0.1	0.01
CG029	69	m	n/a	as	40	−	0.21	0,17	22.3	<0.1	0.02
CG030	65	f	n/a	as	0	−	0.13	0,18	16.3	<0.1	0.02
CG034	55	f	neg	as	0	−	0.09	0,18	23.5	<0.1	0.01
CG035	59	m	neg	as	10	−	0.19	0,2	16.3	<0.1	0.02
CG036	31	m	n/a	as	10	−	0.44	0,23	20.4	<0.1	0.01
CG037	25	f	n/a	as	20	+	0.09	0,17	<3.8	<0.1	0.04
CG041	54	m	n/a	as	20	+	0.3	0.15	12.5	<0.1	0.01
Cohort summary *n* = 42											
			Positive, *n*		26	23	12	12	21	17	16
			Borderline, *n*		n/a	n/a	0	3	6	n/a	n/a
			Negative, *n*		16	19	30	27	15	25	26
			Seropositive		(61.9%)	(54.8%)	(28.6%)	(28.6%)	(50.0%)	(40.5%)	(38.1%)

Results are defined as positive according to the manufacturer’s instructions: OD ratio ≥1.1; AU/ml ≥15; COI ≥1.0; index (S/C) ≥1.4

*AU* arbitrary units; *as* asymptomatic; *COI* cut-off index; *CMIA* chemiluminescent microparticle immunoassay; *CLIA* chemiluminescent immunoassay; *ECLIA* electrochemiluminescence immunoassay; *ELISA* enzyme-linked immunosorbent assay; *f* female; *ID* patient identification; *IFT* immunofluorescence test; *m* male; *n/a* not applicable; *NT* neutralization test; *OD* optical density; *S/C* sample/control; *sym* symptomatic

*Anti-spike

**Anti-nucleocapsid

Our study included the (i) EUROIMMUN(EI)-anti-SARS-CoV-2 IgA and IgG ELISA test, which contains the S1 subunit of the spike protein (EI S1 IgG or EI S1 IgA); (ii) the LIAISON® SARS-CoV-2 S1/S2 IgG CLIA test, containing the S1 and S2 domain of the spike protein (DiaSorin S1/S2 IgG); (iii.)the SARS-CoV-2 IgG CMIA from Abbott detecting anti-nucleocapsid IgG antibodies (Abbott N IgG) and (iv) the Elecsys® anti-SARS-CoV-2 ECLIA test from Roche which uses biotinylated and ruthenylated nucleocapsid antigen for the determination of antibodies against SARS-CoV-2 (Roche N Ab). For comparison, test results from these commercially available assays were evaluated in relation to the previously described in-house NT.

Of the 26 sera that were tested positive by NT, 12 were also tested positive with the EI S1 IgG or IgA assay, while all 16 NT negative sera have been identified as negative. Of note, 10 of the 26 individuals were positive in the EI S1 IgG as well as the EI S1 IgA test (Table 1). Although the DiaSorin S1/S2 IgG test identified 16 of the 26 NT-positive individuals as positive, 5 of the 16 NT negative individuals were tested positive as well. The Abbott N IgG test detected 16 positive individuals while the Roche N Ab test determined 17 of the 26 NT-positive individuals as positive. In both tests, none of the NT negative sera was above the respective cut-off. Thus, the negative agreement between the NT and EI S1 IgG or IgA test, the Roche N Ab assay, and the Abbott N IgG test was 100%. However, the false-positive rate of the DiaSorin S1/S2 IgG assay was 31.3%.

Taking the performed in-house NT as standard, the EI S1 IgG or IgA test had the lowest sensitivity (46.2%, 95% CI [0.355-0.712]; IgA and/or IgG positive 53.8%). The sensitivity of the Abbott N IgG assay as well as the DiaSorin S1/S2 IgG test was 61.54% (95% CI [0.425-0.776]) in relation to NT results. Notably, the Roche N Ab assay had the highest sensitivity with 65.4% (95% CI [0.462-0.806]) (Table 2). Taken together, the N-restricted tests showed a better sensitivity compared to the S-restricted tests. Nevertheless, the use of the commercially available automated serological test systems described herein would result in the reporting of a lower seroprevalence compared to the in-house neutralization test.

**Table 2:** Performance characteristics of the EUROIMMUN, DiaSorin, Roche, and Abbott SARS-CoV-2 antibody platforms, Heinsberg District, Germany, April 2020 (*n* = 26)

		EUROIMMUN			DiaSorin	Roche	Abbott
		S1 IgA	S1 IgG	S1 IgA and/or IgG	S1/S2 IgG	N antibodies	N IgG
Overall NT positive ≥20	n/N	12/26	12/26	14/26	16/26	17/26	16/26
	Value	0.462	0.462	0.538	0.615	0.654	0.615
	(95% CI)	0.288-0.645	0.288-0.645	0.355-0.712	0.425-0.776	0.462-0.806	0.425-0.776

For sensitivity calculations of the commercial assays, only the NT-positive samples (≥20) were used

*CI* confidence interval; *N* nucleocapsid; *NT*: neutralization test; *S* spike

### Correlation of commercial SARS-CoV-2 antibody assay results with neutralization ability

**Fig. 2:**
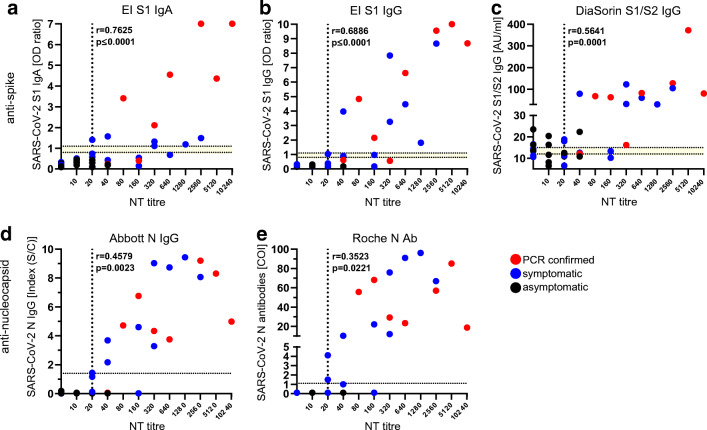
Correlation between commercial SARS-CoV-2 antibody tests and the neutralization titer, Heinsberg District, Germany, April 2020 (*n* = 42). AU: arbitrary units; COI: cut-off index; EI: EUROIMMUN; N: nucleocapsid; NT: neutralization test; OD: optical density; *r*: correlation coefficient; S1: spike domain 1; S2: spike domain 2; S/C: sample/control; SARS-CoV-2: severe acute respiratory syndrome coronavirus 2. The reciprocal of the NT titer is depicted. RT-PCR-confirmed SARS-CoV-2 infections are depicted in red and symptomatic individuals in blue. All asymptomatic individuals are displayed in black. **a** and **b** EUROIMMUN-anti-SARS-CoV-2 IgA and IgG ELISA (Euroimmun). **c** LIAISON® SARS-CoV-2 S1/S2 IgG (DiaSorin). **d** SARS-CoV-2 IgG CMIA (Abbott). **e** Elecsys® anti-SARS-CoV-2 ECLIA test (Roche). The dotted lines indicate the cut-off values recommended by the respective manufacturer to determine positive and negative test results. The borderline area if applicable is indicated in yellow and the vertical line represents the positive cut-off of an NT titer ≥20

To assess which SARS-CoV-2 antibody test platforms are more suitable for predicting neutralizing antibody levels, correlations of commercial SARS-CoV-2 antibody assay results with neutralization test results were determined. The neutralization titer, based on the in-house neutralization test, correlated strongly with all spike antigen-based antibody tests (EI S1 IgA *r* = 0.7625; EI S1 IgG *r* = 0.6886; DiaSorin S1/S2 IgG *r* = 0.5641) (Fig. 2). The weaker correlation of the commercial N-test systems (Abbott N IgG *r* = 0.4579 and Roche N Ab *r* = 0.3523) with the neutralizing antibody titers indicated that S-based systems are more likely to be predictive for functional antibodies.

## Discussion

We assessed and compared the sensitivity of four different available commercial antibody tests EUROIMMUN-anti-SARS-CoV-2 IgA and IgG ELISA, LIAISON® SARS-CoV-2 S1/S2 IgG (DiaSorin) CLIA, the SARS-CoV-2 IgG CMIA from Abbott, and the Elecsys® anti-SARS-CoV-2 ECLIA test from Roche as well as an in-house immunofluorescence and neutralization test, in a SARS-CoV-2 high-prevalence setting. For this, we collected serum samples of close contacts to the NRW index patients at least 6 weeks after possible contact occurred. With respect to serological assays, more than 1 month after a putative infection is sufficient to allow detection of SARS-CoV-2 antibody responses [11, 12]. Various reports suggest that virus-specific IgG levels in positive patients are most reliably detected between 17 days and 8 weeks post infection [12–14].

A peculiarity of this study is that the cohort included 42 individuals who had contact to the NRW index patient at the end of February 2020, a time of uncontained viral spread since health authorities had not yet taken containment measures. Although only 8 individuals were previously tested positive for SARS-CoV-2 by PCR, we found that 26 of the 42 individuals had neutralizing antibodies (61.9%) in an in-house neutralizing test (NT). This high seroprevalence is consistent with data from a high school in France describing that 40.9% of pupils, teachers, and the school staff combined had SARS-CoV-2 antibodies [15].

The study by Streeck et al. [16], sampling a random cohort of 1007 people from the area where the German Heinsberg outbreak occurred, found an anti-SARS-CoV-2 seroprevalence in the range of 15%. With respect to this lower seroprevalence reported by Streeck et al., it is important to acknowledge the different sampling approaches. Nevertheless, our data suggest that in such a high-prevalence setting, a substantial number of convalescent COVID-19 cases may be missed with commercial serological assays. Although we found a high concordance of immunofluorescence test (IFT) positive with NT-positive individuals (23 of 26; 88.5% 95% CI [0.710-0.960]), the commercially available SARS-CoV-2 antibody assays from four companies evaluated in this study had led to fewer positive test results, suggesting a lower sensitivity compared to the NT or the IFT in this cohort. These results are in line with Kohmer et al. [17]. Furthermore, this adds to the difference observed between our study and the one of Streeck et al. as only ELISA IgG seropositive sera were analyzed in their NT [16].

Although none of the commercial assays detected more than 65.4% of SARS-CoV-2 NT-positive individuals, we see a slightly higher sensitivity of nucleocapsid assays compared to assays using spike, which is in line with previous findings [18]. However, since the median time of sera sampling after symptom onset was 43 days, this could not be attributed to an earlier anti-N response as described by Grzelak et al. [19]. Notably, both N-restricted assays gave no false-positive results in our small cohort even though a higher cross reactivity to human coronaviruses (HCoVs) has been proposed [5]. The sensitivity of the assays as reported by the manufacturers ranged between 93.8 and 100% ≥14 to >21 days post symptom onset. However, critical COVID-19 cases seem to mount a more robust antibody response than non-critical hospitalized patients [11]. Accordingly, all assays detected higher antibody levels in the 8 confirmed PCR-positive cases, a group that showed a more severe disease course than the other groups. It is important to note that the sensitivities calculated in the current study refer to a high-prevalence setting with mild and asymptomatic courses and only one non-critical hospitalized patient. In turn, the sensitivity might be insufficient for detection of all mild or asymptomatic cases as in this cohort. A study performed in South Korea found that serological testing of PCR confirmed but asymptomatic patients only identified 71% positive individuals, while neutralizing antibodies were detectable in all asymptomatic individuals [13].

In line with previous studies, ELISA and CLIA assays detecting anti-S or anti-N antibodies had a mild to strong correlation with neutralization titers [10, 20]. The EUROIMMUN-anti-SARS-CoV-2 IgA and IgG ELISA tests showed the strongest correlation with antibody function (IgA *r* = 0.7625, *p* ≤ 0.0001; IgG *r* = 0.6886, *p* ≤ 0.0001) followed by the LIAISON® SARS-CoV-2 S1/S2 IgG assay (*r* = 0.5641, *p* = 0.0001). In the current study, serological assays detecting spike antibodies showed better correlations, which might be due to the fact that the spike protein is the major target for neutralizing antibodies for related coronaviruses and proposedly as well for SARS-CoV-2 [21, 22]. Wu and colleagues as well report that the neutralizing antibody titers correlate with spike-binding antibodies which target the viral S1, RBD, and S2 regions [23].

Of note, the DiaSorin S1/S2 IgG assay rendered five false-positive results from NT assay-negative samples. This finding might suggest a cross reactivity to other endemic HCoVs, possibly because the spike S2 subunit is more conserved among HCoVs than the S1 domain, but this needs to be confirmed with further experiments [10, 20]. Since neutralizing antibody titers in SARS-COV-2-infected individuals varied widely, the EUROIMMUN-anti-SARS-CoV-2 IgA and IgG assay could be considered for pre-screenings to determine optimal donors for convalescent plasma or estimating the induction of virus-specific neutralizing antibodies after vaccination.

Calculation of sensitivity of all commercially available test systems was performed with the NT as reference for past SARS-CoV-2 infection. As 5.7% of hospitalized COVID-19 patients do not generate neutralizing antibodies neither at the time of discharge nor thereafter [23], we could not exclude the possibility that we potentially missed some SARS-CoV-2-infected individuals. Moreover, since we included volunteers from a high-risk area in this small sample study, the data might not be representative for a low-prevalence setting, which is the current situation in most areas of Europe.

In conclusion, the four commercially available high-throughput assays for the detection of SARS-CoV-2-specific antibodies differed in their sensitivity and their potential to predict the neutralization capacity of patient sera. The N-immunoassays tested here seemed to be more sensitive compared to S1 spike protein assays. However, sensitivity of the here described commercial SARS-CoV-2 antibody assays was insufficient for detection of all individuals that were shown to have neutralizing anti-SARS-CoV-2 antibodies. These results should be considered in future population-based seroprevalence studies.

## Supplementary Information

ESM 1 (PDF 447 kb)

## Data Availability

The data supporting the findings of this study are available within the article and its supplementary material.
