# Supplementary material for: Sensitivity of anti-SARS-CoV-2 serological assays in a high-prevalence setting
Source: Eur J Clin Microbiol Infect Dis. 2021 Feb 3;40(5):1063–71. doi: 10.1007/s10096-021-04169-7 (PMC7856849; doi:10.1007/s10096-021-04169-7)
Supplement: Supplementary file 1 — (PDF 447 kb) [file 10096_2021_4169_MOESM1_ESM.pdf]

Table S1: Patient characteristics, Heinsberg District, Germany, April 2020 (n=42)

	Complete cohort		Asymptomatic		Symptomatic	
Number of individuals n, (%)	42	(100%)	16	(38.1%)	26	(61.9%)
Age, median (range)	44	(18-70)	50	(23-70)	42	(18-59)
Gender, n (%)						
Female	26	(61.9%)	16	(61.5%)	10	(38.5%)
Male	16	(38.1%)	10	(62.5%)	6	(37.5%)
Previous PCR test history n (%)						
SARS-CoV-2 PCR positive	8	(19.0%)	1	(12.5%)	7	(87.5%)
SARS-CoV-2 PCR negative	18	(42.9%)	6	(33.3%)	12	(66.7%)
Not tested	16	(38.1%)	9	(56.3%)	7	(43.8%)
Symptoms, n (%)						
Sore throat					18	(69.2%)
Fatigue					13	(50%)
Joint and muscle pain					18	(69.2%)
Abdominal pain					1	(3.8%)
Diarrhoea					6	(23.1%)
Cough					17	(65.4%)
Fever					10	(38.5%)
Loss of taste					6	(23.1%)
Loss of smell					4	(15.4%)
Shortness of breath or difficulty of breathing					8	(30.8%)
Headache					16	(61.5%)

Table S2: IFT and NT results in PCR confirmed, symptomatic and asymptomatic individuals, Heinsberg District, Germany, April 2020 (n=42)

	Complete cohort		PCR confirmed cases		PCR negative or not tested: Asymptomatic		PCR negative or not tested: Symptomatic	
Number of individuals n, (%)	42	(100%)	8	(19.0%)	15	(35.7%)	19	(45.2%)
IFT (n=42) n (%)								
Positive	23	(54.8%)	8	(100%)	3	(20.0%)	12	(63.2%)
Negative	19	(45.2%)	0	(0%)	12	(80.0%)	7	(36.8%)
Neutralisation assay (n=42)								
Positive ≥20, n (%)	26	(61.9%)	8	(100%)	5	(33.3%)	13	(68.4%)
(NT range)	(20-10,240)		(40-10,240)		(20-2,560)		(20-40)	
Negative <20, n (%)	16	(38.1%)	0	(0.0%)	10	(66.6%)	6	(31.6%)

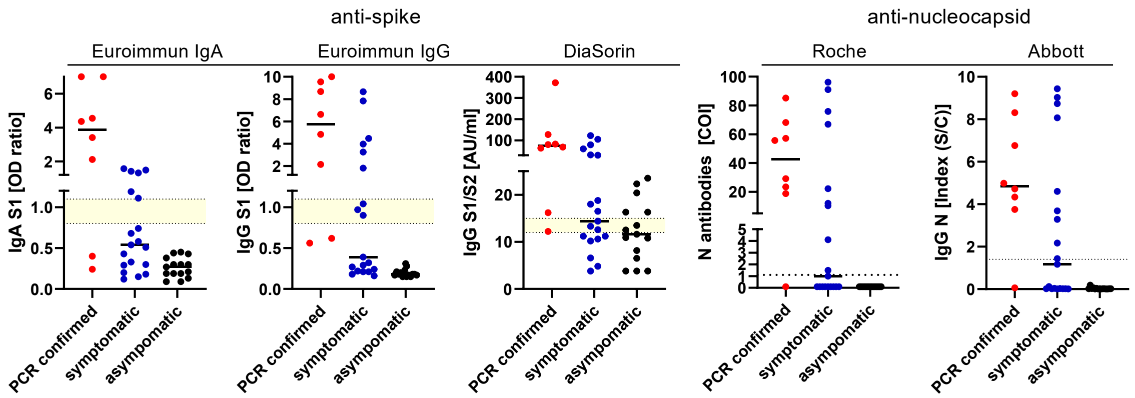

Figure S1: Detection of SARS-CoV-2 specific antibodies with commercial high-throughput Anti-SARS-CoV-2 assay systems, Heinsberg District, Germany, April 2020 (n=42)

AU: arbitrary units; COI: cut-off index; N: nucleocapsid; OD: optical density; S: spike; S/C sample/control

Eight individuals with PCR-confirmed SARS-CoV-2 infection (red), 19 symptomatic (blue) and 15 asymptomatic individuals (black). IgA and IgG S1 Anti-spike antibodies detected by ELISA from EUROIMMUN and IgG S1/S2 CLIA DiaSorin as well as the two tested nucleocapsid-directed antibody tests from Roche and Abbott. Borderline areas of the EUROIMMUN ELISA and the DiaSorin CLIA are indicated in yellow. Dotted lines represent the cut-off value for each assay.

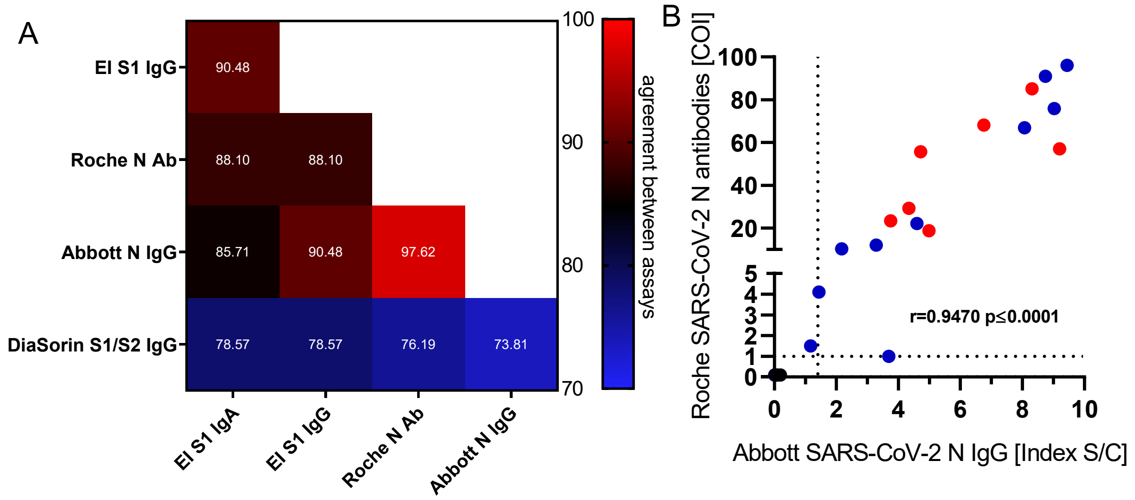

Figure S2: Agreement between commercially available Anti-SARS-CoV-2 spike and nucleocapsid assays, Germany Heinsberg District, April 2020 (n=42)

COI: cut-off index; EI: EUROIMMUN; N: nucleocapsid; r: correlation coefficient; SARS-CoV-2: severe acute respiratory syndrome coronavirus 2; S: spike; S/C: sample/control

(A) The comparability between each assay system was calculated and depicted within the heatmap. (B) Correlation between the Roche N Ab test and the Abbott N IgG test, the two N-antibody assays (r=0.9470, p≤0.0001). All results were colour coded according to their status in PCR confirmed individuals (red), symptomatic (blue) and asymptomatic (black).
